# Dual roles of *Drosophila* reward-encoding dopamine neurons in regulating innate and learned behaviors

**DOI:** 10.1016/j.isci.2025.113817

**Published:** 2025-10-23

**Authors:** Fiorella V. Lozada-Perdomo, Yuzhen Chen, Ruby V. Jacobs, Joyce Yeo, Meifeng (Maia) Yang, Janhavi Bhalerao, Anita V. Devineni

**Affiliations:** 1 Department of Biology, Emory University, Atlanta GA 30322, USA; 2 Neuroscience Graduate Program, Emory University, Atlanta GA 30322, USA

**Keywords:** Neuroscience, Behavioral neuroscience

## Abstract

Dopaminergic neurons (DANs) play a key role in learning the value of cues that predict reward. The fruit fly *Drosophila* has provided a powerful model to dissect the mechanisms by which reward-encoding DANs mediate reward learning. However, the role of these DANs in regulating innate behaviors is less clear. Here, we show that activating the entire population of reward-encoding DANs in *Drosophila* drives acute aversion in multiple behavioral assays, including feeding, locomotion, and spatial preference, even as these neurons confer a positive value onto associated cues to promote future learned attraction. Activating subsets of DANs reveals that the effects on innate and learned behaviors are dissociable. Based on the known circuitry, it is likely that immediate aversion and learned attraction mediated by DANs arise from distinct effects – direct activation versus synaptic plasticity – on the same target neurons. These results reveal distinct roles for reward-encoding DANs in guiding immediate and future behavior.

## Highlights

• Reward-encoding dopamine neurons (DANs) are known to mediate appetitive learning
• Surprisingly, the activation of reward DANs causes immediate aversion in several assays
• The opposing effects of DANs on innate and learned behaviors are dissociable
• The opposing effects likely arise from distinct effects on the same target neurons

## Graphical abstract

**Figure:**
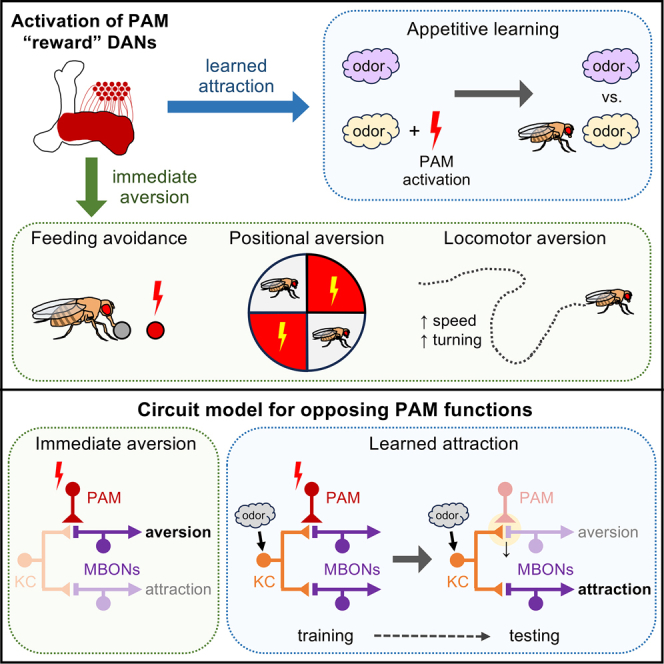

## Introduction

The ability to seek out and respond to rewarding stimuli in the environment, such as food, water, and potential mates, is critical for survival. To do so, brains have evolved “reward systems” that are activated by rewarding stimuli and reinforce reward-seeking behaviors.^1^ In mammals, the mesolimbic dopaminergic pathway represents one of the brain’s most well-known reward systems. The release of dopamine provides a reward signal that promotes learning, enabling an organism to learn the value of cues that predict a reward.^2^^,^^3^^,^^4^ In addition, dopamine invigorates ongoing behaviors, potentially representing a motivation signal.^2^ Food consumption is a prominent example of an immediate behavior that is modulated by dopamine signals. Dopaminergic neurons (DANs) in the mesolimbic system are activated by highly palatable foods and promote eating, and the dysregulation of dopamine signaling may contribute to overeating and obesity.^5^^,^^6^

DANs also represent a major reward system in invertebrates, including the fruit fly *Drosophila melanogaster*.^1^ As in mammals, different populations of DANs in *Drosophila* respond to stimuli of positive or negative valence, and the protocerebral anterior medial (PAM) cluster of DANs has emerged as a central node for reward encoding.^1^ These DANs innervate the mushroom body, the learning and memory center of the fly brain, and their role in appetitive learning has been well established.^7^ PAM DANs are activated by natural rewards, such as sugar or water, and are necessary for appetitive olfactory learning, in which pairing a reward with a neutral odor causes future attraction to the paired odor.^8^^,^^9^^,^^10^^,^^11^ Moreover, the artificial activation of PAM DANs can substitute for reward and cause learned attraction to a stimulus that was previously paired with PAM activation.^8^^,^^9^^,^^12^^,^^13^ Thus, a major function of PAM DANs is to confer a rewarding value onto associated stimuli to guide future reward seeking. PAM DANs also provide reward-related signals for other types of learning beyond classical appetitive conditioning, such as learning the relative values of aversive stimuli or promoting the extinction of an aversive memory.^14^^,^^15^^,^^16^ Although different subsets of PAM DANs can have different functional roles, such as driving short-term versus long-term memory, most subsets encode reward-related signals and promote appetitive learning.^9^^,^^17^^,^^18^

While many studies have established the role of PAM DANs in learning, the role of PAM DANs in regulating innate behaviors – behaviors that do not require learning – is less clear. In behavioral assays where PAM DANs cause appetitive learning, they do not necessarily promote innate attraction. For example, pairing PAM activation with salt did not affect immediate preference for salt during the activation period but caused a learned preference for salt (in the absence of DAN activation) when flies were tested 10 min or 24 h later.^13^ Through neuronal silencing or activation experiments, subsets of PAM DANs have been implicated in promoting food seeking,^19^^,^^20^ sugar feeding,^21^ water seeking,^10^ and odor tracking.^22^ These studies suggest that the activity of PAM DANs may invigorate innate reward-seeking behaviors, similar to the role of dopamine in mammals. However, other studies have instead suggested that PAM DANs may have an aversive role in immediate behavior, such as promoting avoidance of an aversive odor^23^ or diminishing learned attraction,^24^ or may have differing effects depending on the PAM subset or the behavioral assay.^25^^,^^26^

In this study, we initially set out to investigate how PAM DANs influence feeding, an innate behavior whose regulation by DANs has not been well studied. While we hypothesized that activating PAM DANs would promote feeding, we found the opposite: the activation of the PAM population strongly suppressed feeding under a variety of conditions. This result prompted us to test the role of PAM DANs in other innate behaviors, including locomotion and positional preference. Our results show that although the activation of the PAM population robustly promotes appetitive learning, as previously established, this activation is innately aversive to flies and drives aversive locomotor and feeding responses. Activating PAM subsets caused appetitive learning but not immediate aversion, suggesting that aversion may result from a subset that we did not test or from the combined activation of the entire PAM population. These results reveal a sharp dichotomy between the role of PAM DANs in regulating innate versus learned behaviors. Based on the known circuitry of the mushroom body, these two effects likely arise from different actions – direct activation versus synaptic plasticity – on the same target neurons.

## Results

### Both silencing and activation of PAM dopamine neurons reduce feeding

**Figure 1:**
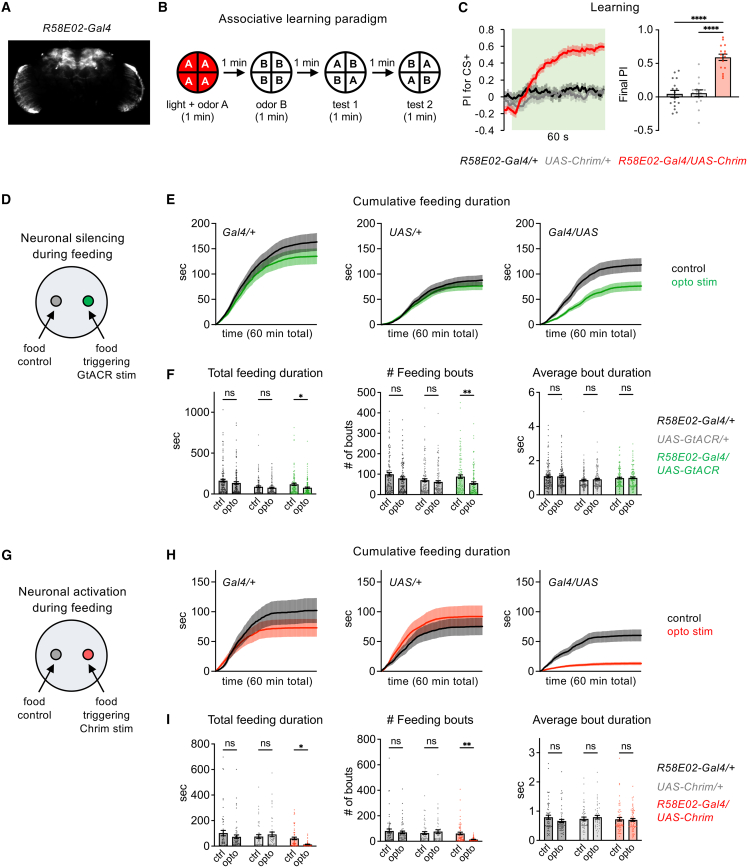
Both activation and silencing of the entire PAM DAN population reduce feeding (A) Expression pattern of *R58E02-Gal4* driving *UAS-Chrimson.* (B) Schematic of the associative learning assay, showing odor A as the CS+ and odor B as the CS-. Learned preference is quantified by the preference index (PI), defined as (# flies in the CS+ quadrants – # flies in the CS- quadrants)/total # flies. Data from test 1 and test 2 are combined. (C) PAM activation using *R58E02-Gal4* driving *UAS-Chrimson* elicits appetitive learning, as quantified by the PI for the CS+ over time (left) or final PI (right) (*n* = 16 trials, 8 sets of flies). Genotypes were compared using one-way ANOVA followed by Dunnett’s post-tests. In all figures showing learning experiments, green shading indicates odors on, and the final PI represents the average PI over the last 5 s, unless otherwise specified. In the line graph, experimental flies may appear to show a negative PI before odor onset because of the repeated tests that are combined (see panel B); after the first test, some flies continue to reside in the CS+ quadrants, which become the CS- quadrants for the second test. (D–I) Both PAM silencing (panels D–F, *n* = 117–123 flies) and activation (panels G-I, *n* = 49–60 flies) decrease sugar feeding in the optoPAD. Line graphs (E, H) show cumulative feeding duration over a 60 min assay on control vs. optogenetic-linked food sources (100 mM sucrose) for each genotype. Bar graphs (F and I) show total values for various feeding metrics over the entire assay. Control and opto values were compared by two-way repeated measures ANOVA followed by Bonferroni’s post-tests. See Figure S1 for representative plots of feeding activity over time. For all figures: Flies carrying only the Gal4 (*Gal4/+*) or UAS (*UAS/+*) transgenes are used as controls. When comparing metrics across genotypes, experimental flies must significantly differ from both controls in order to show an effect. All graphs represent mean ± SEM, and points represent individual flies or trials. ∗∗∗∗*p* < 0.0001, ∗∗∗*p* < 0.001, ∗∗*p* < 0.01, and ∗*p* < 0.05, ns = not significant (*p* > 0.05).

Given the known role of PAM DANs in encoding reward signals, we asked how the activity of PAM DANs influences sugar feeding. We targeted the entire population of PAM DANs using *R58E02-Gal4*, a well-characterized and highly specific line that has been used in many previous studies.^8^^,^^9^^,^^10^^,^^14^^,^^15^^,^^27^ We verified the expression pattern of *R58E02-Gal4* using immunostaining (Figure 1A), confirming that the only labeled cells other than PAM DANs are cells in the optic lobe that were reported to be glia.^8^ In earlier studies^28^^,^^29^ we confirmed that PAM DANs are activated when flies taste sugar, as previously reported.^8^^,^^22^ In addition, we verified that optogenetic activation of PAM DANs using *R58E02-Gal4* to express *UAS-Chrimson*, encoding a light-gated cation channel,^30^ causes robust appetitive learning (Figures 1B and 1C). In our learning assay (Figure 1B), the conditioned odor (CS+) is presented for 1 min along with PAM activation, followed by 1 min presentation of a control odor (CS-) without neuronal activation, and flies then choose between the CS+ and CS-. We observed a strong preference for the CS+ (Figure 1C), as expected based on the known role of PAM DANs in associative learning.^8^^,^^9^^,^^12^^,^^13^

Having verified the role of PAM DANs in appetitive learning, we then tested whether their activity regulates sugar feeding. We measured feeding in the optoPAD, which uses a capacitance sensor to detect feeding events and thus provides a high-resolution quantification of feeding behavior.^31^^,^^32^ We used closed-loop light stimulation to optogenetically activate or silence PAM DANs whenever the fly interacted with one food source (100 mM sucrose), while a second identical food source not linked to optogenetic stimulation served as the control. The closed-loop paradigm was designed to alter PAM activity during times when PAM DANs would normally be activated by sugar taste, thus mimicking a scenario where sugar stimulation causes more or less PAM activity than usual. We note that this assay may elicit operant conditioning, as the fly’s actions (feeding) are reinforced by neuronal activation or silencing, although this was not the goal of the paradigm. For all feeding experiments, flies were food-deprived for one day (unless otherwise specified) to ensure that they were motivated to consume sugar, and the same conditions were used for other behavioral experiments described later.

We first silenced PAM DANs by using *R58E02-Gal4* to express *UAS-GtACR*, encoding a light-gated chloride channel,^33^ and analyzed feeding behavior. Feeding events in the optoPAD are clustered into short feeding bouts that last for up to a few seconds, with dozens to hundreds of bouts occurring over the course of 1 h (Figure S1).^32^ PAM silencing reduced the number of feeding bouts and total feeding duration (Figures 1D–1F), implying that PAM activity normally promotes sugar feeding. Feeding bout duration was not affected (Figure 1F), suggesting that PAM activity influences the decision to initiate the next feeding bout rather than determining the length of an individual bout.

To determine whether increasing PAM activity could increase sugar feeding, we activated PAM DANs using *R58E02-Gal4* driving *UAS-Chrimson*. Surprisingly, PAM activation strongly suppressed feeding, showing an even larger effect than PAM silencing (Figures 1G–1I). PAM activation suppressed the number of feeding bouts and the total feeding duration but did not affect the duration of individual bouts, similar to PAM silencing (Figure 1I). Thus, while PAM silencing decreases sugar feeding, suggesting that reward signals encoded by PAM DANs are required to promote normal levels of feeding, activating PAM DANs suppresses rather than enhances feeding.

### Activation of PAM dopamine neurons suppresses feeding across a variety of conditions

**Figure 2:**
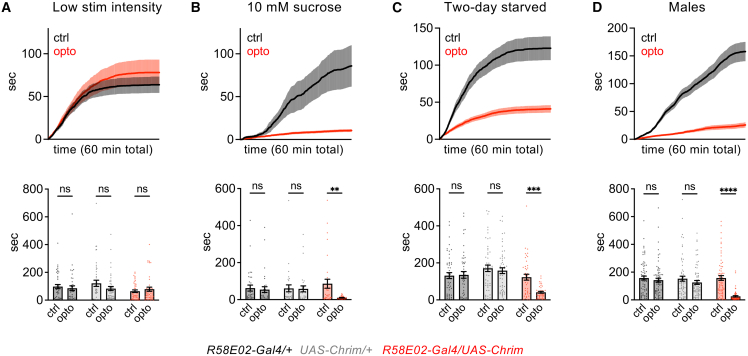
Activation of PAM DANs suppresses feeding under a variety of conditions The effect of PAM DAN activation (*R58E02-Gal4* driving *UAS-Chrimson*) on feeding was measured under 4 different conditions. Experiments used female flies starved for one day with 13.5 μW/mm^2^ light activation and 100 mM sucrose but varied one parameter at a time, using low intensity (3.8 μW/mm^2^) light stimulation in panel A (*n* = 41–44 flies), 10 mM sucrose in panel B (*n* = 30–33 flies), two-day starvation in panel C (*n* = 43–55 flies), and male flies in panel D (*n* = 66–70 flies). Top graphs show the cumulative feeding duration over 60 min for experimental flies (*R58E02-Gal4/UAS-Chrim*) on control and optogenetic-linked food sources. Bottom graphs show the total feeding duration over 60 min for experimental and control genotypes. Feeding on control vs. opto food was compared by two-way repeated measures ANOVA followed by Bonferroni’s post-tests. ∗∗∗∗*p* < 0.0001, ∗∗∗*p* < 0.001, ∗∗*p* < 0.01, ∗*p* < 0.05, ns = not significant (*p* > 0.05).

We repeated PAM activation in the optoPAD under several different conditions to assess how consistent and robust the feeding suppression was. First, we tested activation using a lower light intensity. The light intensity used in our initial experiments was 13.5 μW/mm^2^, which is already a relatively low intensity compared to the range of intensities used for Chrimson activation in other studies,^13^^,^^26^^,^^34^ but we wondered whether an even lower intensity might lead to a more modest increase in PAM activity that could increase rather than decrease feeding. When we lowered the light intensity to 3.8 μW/mm^2^, we did not observe a significant effect on sugar feeding (Figure 2A). Thus, the feeding suppression effect arises between ∼4 and 13 μW/mm^2^ stimulation, and we do not observe evidence of feeding enhancement.

Second, we tested whether PAM activation also suppresses feeding at lower concentrations of sugar, which would be expected to elicit lower endogenous activation of PAM DANs. PAM activation strongly suppressed feeding at 10 mM sucrose (Figure 2B), similar to the effect at 100 mM. Third, we tested flies that were starved for two days rather than one day, which should increase the flies’ drive to consume sugar. Again, PAM activation strongly suppressed feeding on 100 mM sucrose (Figure 2C). Finally, we tested male flies, since our initial experiments used females, and we found that PAM activation strongly suppressed feeding in males as well (Figure 2D). These experiments show that activating the population of PAM DANs causes strong feeding suppression across different sugar concentrations, hunger states, and sexes.

### Activation of PAM dopamine neurons causes aversion in multiple behavioral assays

We next asked whether the activation of PAM DANs causes acute aversion in other behavioral assays. The optoPAD quantifies the feeding behavior of freely moving flies choosing between two food sources, so feeding suppression could reflect a decrease in feeding initiation or locomotor changes causing the fly to leave the food source. Decreased feeding in the optoPAD could also be due to aversive learning or operant conditioning if flies learn to avoid the food source paired with light, but this possibility seems less likely given that flies display learned attraction, rather than aversion, to stimuli paired with optogenetic PAM activation (Figure 1C).^13^

**Figure 3:**
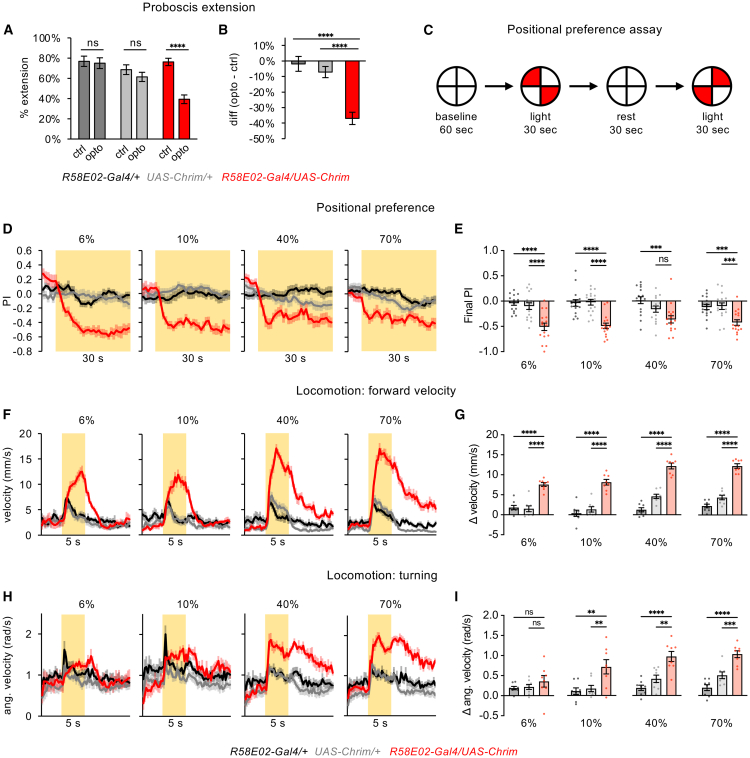
Activation of PAM DANs causes acute aversion in multiple behavioral assays (A and B) PAM activation using *R58E02-Gal4* driving *UAS-Chrimson* suppressed proboscis extension to 100 mM sucrose (*n* = 32–85 flies). (A) Percentage of trials eliciting proboscis extension in the presence or absence of light stimulation (paired t-tests). (B) Difference in the percentage of trials causing proboscis extension with and without light stimulation (one-way ANOVA followed by Dunnett’s test). (C) Schematic of the positional preference assay. Preference for the light quadrants was quantified by the PI, calculated as (# flies in light quadrants - # flies in dark quadrants)/total # of flies. Data for the two tests are combined. (D–E) Flies show positional aversion to PAM activation using *R58E02-Gal4* driving *UAS-Chrimson* (*n* = 16 trials, 8 sets of flies). (D) PI over time. Experimental flies may appear to show a positive PI before light onset because of the repeated tests that are combined (see panel C); after the first test, flies may continue to avoid the previously illuminated quadrants until the next test. For all figures, yellow shading indicates light on. (E) Comparison of final PI (one-way ANOVA followed by Dunnett’s test). Final PI for all preference figures represents the average PI over the last 5 s, unless otherwise stated. (F–I) PAM activation using *R58E02-Gal4* driving *UAS-Chrimson* (*n* = 8 sets of flies) caused a strong increase in forward velocity (F-G) and turning (H-I). Line graphs show forward (F) or angular (H) velocity over time; bar graphs show the change in forward (G) or angular (I) velocity during the light period as compared to the pre-light baseline period (one-way ANOVA followed by Dunnett’s test). ∗∗∗∗*p* < 0.0001, ∗∗∗*p* < 0.001, ∗∗*p* < 0.01, ∗*p* < 0.05, ns = not significant (*p* > 0.05). See Figure S2 for additional experiments confirming the behavioral effects of PAM activation.

We first tested how PAM activation with *R58E02-Gal4* affects proboscis extension, a motor response that represents the initiation of feeding. Proboscis extension is tested in immobilized flies that are stimulated with sugar, providing a readout of feeding initiation that is independent of locomotor changes. We compared proboscis extension to sugar in the presence and absence of optogenetic PAM activation, with the onset of light stimulation occurring immediately before sugar delivery (unlike the optoPAD assay, where light onset occurs immediately after feeding onset, and differing from operant conditioning paradigms). Each fly was given a single set of light stimulation and control (non-light) trials, with control trials occurring first, in order to minimize any effect of learning. We found that PAM activation suppressed proboscis extension to sugar (Figures 3A and 3B), representing an aversive effect that may contribute to feeding suppression in the optoPAD.

We then tested whether flies show preference or aversion for optogenetic PAM activation with *R58E02-Gal4* using a positional preference assay. We presented red light in two opposing quadrants of a circular arena and asked whether flies would prefer or avoid residing in the light quadrants, where they experience PAM activation (Figure 3C). Experimental flies showed consistent light aversion across a wide range of light intensities (Figures 3D and 3E). These results imply that flies find PAM activation to be aversive despite the ability of PAM activation to cause appetitive learning.

Finally, we quantified how PAM activation affects locomotion by presenting light throughout the arena for 5 s. The activation of known aversive neurons, such as bitter-sensing neurons, causes flies to immediately turn and increase their forward speed, behaviors that would normally help them navigate away from the stimulus.^35^ Similar to bitter neuron activation, PAM activation caused a strong increase in both forward and angular velocity compared to controls, representing an aversive response (Figures 3F–3I). However, we observed notable differences from the locomotor effects elicited by bitter neuron activation.^35^ First, while bitter neuron activation causes flies to increase their movement during the light stimulus and then immediately stop moving at light offset, as if they are trying to remain in a safe (non-bitter) location, PAM activation caused a sustained increase in locomotion that persisted beyond light offset with no post-light freezing (Figure 3F). Second, bitter stimulation causes only a transient increase in turning (lasting less than 1 s), whereas the increase in turning evoked by PAM activation lasted throughout and beyond the light stimulus period (Figure 3H). Thus, the locomotor responses elicited by PAM DANs are typical of aversive behavior but differ from the responses elicited by bitter neurons.

We performed additional experiments to test the robustness of the acute aversion observed in the positional preference and locomotor assays. First, we repeated these experiments using *R58E02-lexA* driving *lexAop-Chrimson* in order to replicate our findings with different driver and effector transgenes that target the same neuronal population (Figure S2A), and we observed the same results (Figures S2B–S2D). Second, because the preference and locomotor assays used continuous light stimulation for a prolonged period (30 or 5 s, respectively), which might lead to compensatory effects, we repeated these experiments using pulsed light stimulation at 50 Hz. We again observed positional aversion and an increase in forward velocity and turning (Figures S2E–S2G), consistent with the effects of continuous light (Figures 3D–3I). Finally, we tested fed flies rather than one-day starved flies and observed the same effects (Figures S2H–S2J), indicating that these aversive responses do not require a food-deprived state.

Together, these results show that activating the population of PAM DANs causes acute aversion across several behavioral assays despite their role in appetitive learning. Some of these responses may be related, as decreased feeding in the optoPAD may reflect decreased proboscis extension to sugar and increased walking and turning that would drive the fly away from the food source. However, some of the aversive responses are independent; for example, decreased proboscis extension measured in immobilized flies does not depend on locomotor changes.

### Activation of certain PAM neuron subsets increases feeding

**Figure 4:**
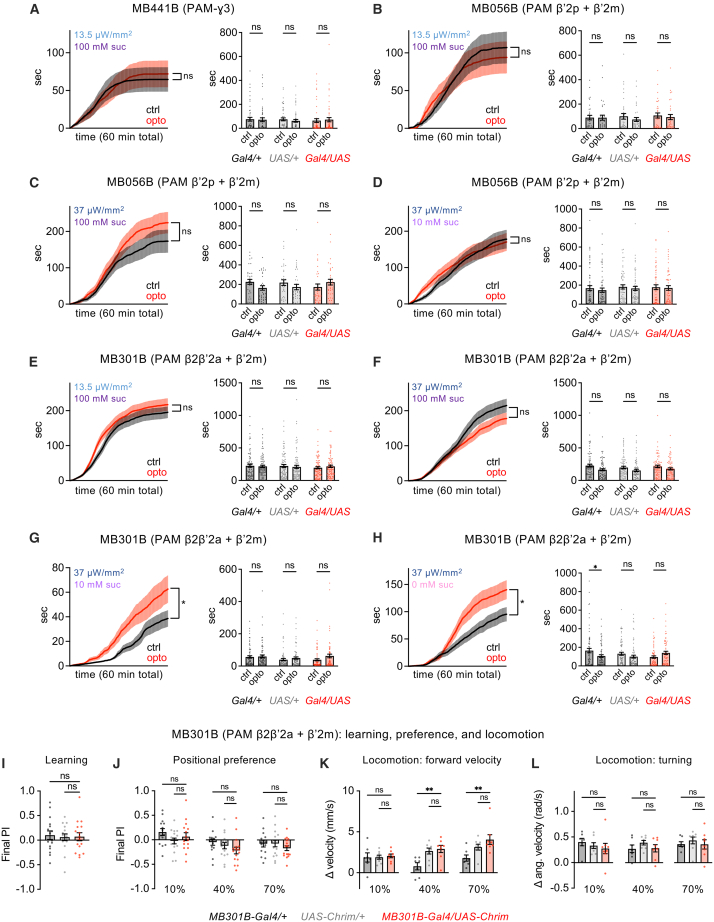
Activation of certain PAM subsets increases feeding (A–H) Activation of certain PAM subsets increases feeding in the optoPAD. Because negative results were observed with our initial protocols (13.5 μW/mm^2^, 100 mM sucrose), additional light intensities or sucrose concentrations were tested for some subsets. Line graphs show cumulative feeding duration over 60 min in experimental flies (*MB-Gal4/UAS-Chrim*), with significance tested using two-way repeated measures ANOVA, and asterisks report significance for the main effect of food type (control vs. opto); note that an interaction between food type and time (*p* < 0.0001) was observed for panels F-H. Bar graphs show total feeding duration over 60 min for flies of each genotype (*MB-Gal4/+, UAS-Chrim/+*, or *MB-Gal4/UAS-Chrim*), and control vs. opto values were compared using two-way repeated measures ANOVA followed by Bonferroni’s post-tests. Sample sizes were 33–60 flies for MB441B or MB056B activation and 69–84 flies for MB301B activation. (I–L) PAM activation using MB301B did not have a significant effect on associative learning (panel I; *n* = 16 trials, 8 sets of flies), positional preference (panel J; *n* = 14–16 trials, 7–8 sets of flies), or forward or angular velocity (panels K-L; n = 7–8 sets of flies) (one-way ANOVA followed by Dunnett’s post-tests). ∗∗∗∗*p* < 0.0001, ∗∗∗*p* < 0.001, ∗∗*p* < 0.01, ∗*p* < 0.05, ns = not significant (*p* > 0.05). See Figure S3 for expression patterns of split-Gal4 lines.

All of the experiments described above were performed using *R58E02-Gal4* or *R58E02-lexA*, lines that target the entire PAM DAN population. Although most subsets of PAM DANs encode reward and promote appetitive learning, the PAM-ɣ3 subset is an exception: it mediates appetitive learning through a suppression of its activity, and activating PAM-ɣ3 causes aversive learning.^36^ We wondered whether PAM-ɣ3 could be responsible for the feeding aversion observed when the entire PAM population is activated, especially because a previous study reported that the activation of PAM-ɣ3 reduced feeding.^21^ We activated PAM-ɣ3 in the optoPAD using the MB441B split-Gal4 line,^37^ whose expression we verified (Figure S3). We did not observe a significant effect of PAM-ɣ3 activation on feeding in our paradigm (Figure 4A), suggesting that the aversive effect of activating the entire PAM population is due to other PAM subsets or the combined effect of multiple subsets.

The same study that tested the effect of PAM-ɣ3 in feeding also identified two subsets of PAM DANs that increased sugar feeding when activated: one subset labeled by MB056B (PAM β′2p + PAM β′2m) and one labeled by MB301B (PAM β2β′2a + PAM β′2m).^21^ We repeated these experiments in the optoPAD (Figures 4B–4H and S3 for verification of expression). While we did not observe significant effects on total feeding time over the entire assay, plotting the time course of feeding revealed a significant (Figures 4G and 4H) or non-significant (Figure 4C) trend toward increased feeding depending on the stimulation intensity and sugar concentration, with the strongest effects observed with MB301B activation at low sugar concentrations. Overall, our results suggest that the activation of some PAM subsets increases feeding, as previously reported,^21^ whereas activating the entire PAM population strongly suppresses feeding.

Because PAM activation using MB301B increased feeding in the optoPAD, we asked whether its activation caused attraction in other behavioral assays. MB301B activation was not able to cause associative learning in our paradigm (Figure 4I) and did not have a significant effect on positional preference (Figure 4J) or locomotion (Figure 4K–4L). Thus, the effects of PAM subsets on different behaviors appear to be dissociable.

### Activation of PAM neuron subsets elicits appetitive learning but not acute aversion

Our results demonstrate that activating the entire PAM DAN population drives acute aversion in several behavioral assays despite causing appetitive learning. This dichotomy could result from different PAM subsets causing each effect or common neurons that cause both effects. To address this question, we asked whether we could find a subset of PAM DANs whose activation causes both appetitive learning and acute aversion. We focused on using positional preference and locomotor assays to test immediate aversive behavior because feeding aversion may reflect a combination of behavioral effects (including learning effects), as discussed above.

**Figure 5:**
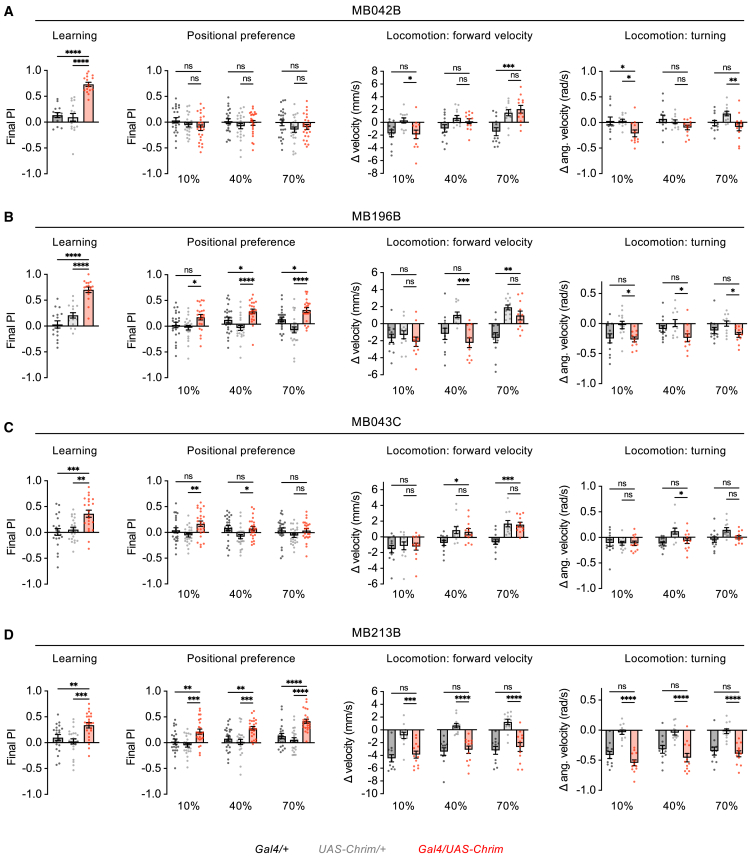
Activation of PAM subsets elicits appetitive learning but not acute aversion (A–D) Effects of activating PAM subsets using 4 different split-Gal4 lines. Each line was tested for associative learning, positional preference, forward velocity, and turning using the assays described in Figures 1 and 3. Genotypes were compared using one-way ANOVA followed by Dunnett’s post-tests. Experimental flies need to differ from both controls in order to show an effect. Sample sizes are 14–24 trials (7–12 sets of flies) for learning, 22–24 trials (11–12 sets of flies) for positional preference, and 11–12 sets of flies for locomotion. ∗∗∗∗*p* < 0.0001, ∗∗∗*p* < 0.001, ∗∗*p* < 0.01, ∗*p* < 0.05, ns = not significant (*p* > 0.05). See Figure S3 for expression patterns of split-Gal4 lines and Figure S4 for graphs showing preference over time.

We used previously characterized split-Gal4 lines to activate four subsets of PAM neurons. Two of the lines, MB042B and MB196B, label several types of PAM DANs, whereas the other two lines, MB043C and MB213B, label only one or two types and have been shown to cause appetitive learning^18^^,^^24^ (Figure S3). We found that PAM activation using all four lines caused appetitive learning, whereas none of them caused aversive effects in either the positional preference or locomotor assays at any light intensity (Figures 5 and S4). In the positional preference assay, MB213B caused a strong preference, MB196B caused a weaker preference, and the other two lines had no significant effect. In the locomotor assay, the only significant effect observed was a small decrease in turning elicited by MB042B at a single light intensity. Together, these data show that, unlike the activation of the entire PAM population, activating subsets of PAM DANs does not generally cause opposite effects on innate and learned behaviors. These findings also show that the regulation of innate and learned preference can be decoupled, as some PAM subsets that cause appetitive learning also elicit acute positional attraction while others do not.

### Aversive effects of PAM neuron activation can override learned or innate attraction

**Figure 6:**
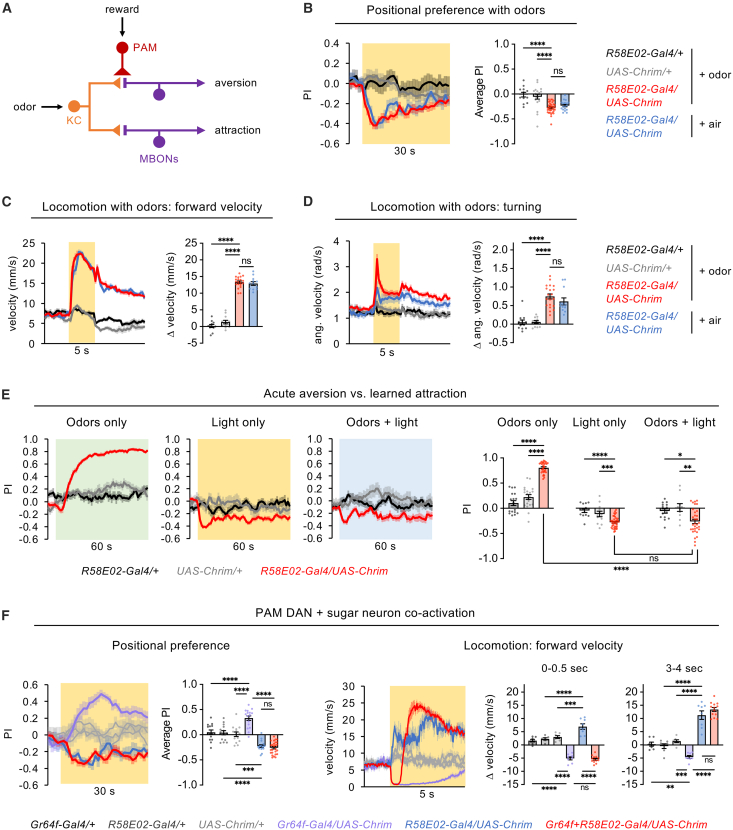
Aversive effects of broad PAM activation can override learned or innate attraction (A) Schematic of the mushroom body circuit. PAM DANs drive appetitive odor learning by causing the depression of KC synapses onto MBONs that promote aversion. PAM DANs may also directly excite the same MBONs to cause immediate aversion. (B) Flies showed positional aversion to PAM activation using *R58E02-Gal4* driving *UAS-Chrimson* in the presence of odors (OCT or MCH) or airflow only (*n* = 12–36 trials, 6–18 sets of flies). 40% light was used. The bar graph quantifies the average PI over the light period. See Figure S5 for OCT and MCH data shown separately. (C and D) PAM activation using 40% light caused an increase in forward (C) or angular (D) velocity in the presence of odors (OCT or MCH) or airflow only (*n* = 12–20 trials, 6–10 sets of flies). Bar graphs quantify the change in velocity during 5 s light compared to the pre-light baseline. See Figure S5 for OCT and MCH data shown separately. (E) Effects of presenting light (50 Hz at 40% intensity) in the CS+ quadrants during the learning test (CS+ vs. CS-) using the associative learning protocol shown in Figure 1B. Graphs show the preference for quadrants containing the CS+ only (left), light only (middle), or both the CS+ and light (right) (*n* = 32–40 trials, 16–20 sets of flies for experimental genotype; *n* = 10–20 trials, 5–10 sets of flies for control genotypes). Bar graphs quantify the average PI over the last 30 s. (F) Effects of co-activating PAM neurons (*R58E02-Gal4*) and sugar-sensing neurons (*Gr64f-Gal4*) on positional preference (*n* = 14–26 trials, 7–13 sets of flies) and locomotion (n = 7–13 sets of flies). Preference was tested using 10% light, an intensity that causes attraction with *Gr64f-Gal4* activation, and the bar graph quantifies the average PI over the last 25 s of the light period. Locomotion data are shown for 40% light; see Figure S5 for effects at 10% and 70%. The velocity graph is zoomed in relative to velocity plots in other figures and data are not smoothed in order to show the transient stopping of flies with co-activation. Locomotion bar graphs quantify the change in stopping at the beginning (first 0.5 s) and middle (3–4 s) of the light period. All 3 control genotypes are shown in light gray on both line graphs. Statistics in these figures represent one-way ANOVA followed by Dunnett’s post-tests (panels B-E) or Bonferroni’s post-tests (panel F). ∗∗∗∗*p* < 0.0001, ∗∗∗*p* < 0.001, ∗∗*p* < 0.01, ∗*p* < 0.05, ns = not significant (*p* > 0.05).

Previous work has shown that PAM DANs promote appetitive olfactory learning by inducing synaptic plasticity in the mushroom body (Figure 6A).^7^ Odors activate specific subsets of Kenyon cells (KCs), which have excitatory synapses onto separate populations of mushroom body output neurons (MBONs) that promote odor attraction or aversion. DANs modulate the strength of KC-MBON synapses, with PAM DANs specifically targeting aversion-promoting MBONs (Figure 6A). When PAM DANs are activated, the release of dopamine depresses the strength of active KC synapses onto aversion-promoting MBONs, thereby ensuring that future presentation of the same odor will lead to attraction by skewing the balance between activation of attractive and aversive MBONs. In addition to causing plasticity at KC-MBON synapses, DANs may also be able to directly excite the MBONs whose synapses they modulate,^24^ and this could explain the aversive behaviors elicited by PAM activation. In this model, PAM activation causes immediate aversion and future learned attraction to paired stimuli through two separate mechanisms that act on the same aversion-promoting MBONs: the direct activation of MBONs causes immediate aversion, whereas the depression of KC-MBON synapses causes future learned attraction. The latter mechanism relies on odor input through KCs, whereas the former does not.

This model raises several questions. First, is the immediate aversion evoked by PAM activation affected by simultaneous activation of KC-MBON synapses, as would occur in a natural environment when odors are present? To address this question, we tested the effect of PAM activation using *R58E02-Gal4* in the presence of background odors. We found that PAM activation still caused strong positional aversion and locomotor enhancement (Figures 6B–6D and S5A–S5C), suggesting that the aversive effects of PAM activation are not affected by the simultaneous activation of KC-MBON synapses.

Second, how will flies behave if the pathways for PAM-mediated learned attraction and immediate aversion are simultaneously activated? To address this question, we elicited associative learning by pairing an odor with PAM activation, but during the learning test, when flies chose between the CS+ and CS-, we also presented light in the CS+ quadrants to activate PAM DANs. We compared these experimental flies to flies that underwent the same associative learning protocol but were only given a choice between the CS+ and CS- odors (the usual learning test) or between the light and dark quadrants (similar to the usual positional preference test, using 50 Hz pulsed light as in Figure S2E). As expected from our earlier results, flies choosing between the CS+ and CS- showed strong attraction to the CS+, and flies choosing between light and dark quadrants showed aversion to the light quadrants (Figure 6E), although this aversion seemed weaker than in previous experiments without airflow (Figure S2E). When the CS+ quadrants also included light, the CS+ preference disappeared and the flies showed a similar level of aversion to the light quadrants as with light alone. (Figure 6E). Thus, acute aversion elicited by PAM activation, likely through direct connections to MBONs, can completely override learned attraction elicited by synaptic depression of KC-MBON synapses.

Finally, can acute aversion driven by PAM DANs override innate attraction elicited by other pathways, such as those activated by sweet taste? To address this question, we co-activated sugar-sensing taste neurons along with PAM DANs. We previously showed that sugar neuron activation causes positional preference (at low light intensities) and strong locomotor suppression,^38^ opposite to the effects of PAM activation. When sugar neurons and PAM DANs were co-activated during the positional preference assay, flies showed an aversion to the light quadrants that was similar to the level of aversion elicited by PAM activation alone (Figure 6F). When sugar neurons and PAM DANs were co-activated during the locomotor assay, a combination of behaviors was observed: flies stopped transiently, similar to sugar activation alone, and then increased their forward speed well beyond baseline levels, similar to PAM activation alone (Figures 6F and S5D). A similar behavioral sequence was also observed when co-activating sugar- and bitter-sensing neurons,^38^ suggesting that cross-inhibition between pathways for locomotor stopping and running may enable a “winner take all” model where one of the two pathways dominates at each time rather than causing an intermediate behavior, and which pathway dominates switches partway through light stimulation. These results show that acute aversion elicited by PAM DANs overrides positional preference elicited by the sugar pathway and overrides locomotor stopping during certain time periods.

Together, these results show that PAM activation drives acute aversion in either the presence or absence of odor-evoked activation of KC-MBON synapses, and PAM-driven aversion overrides learned attraction mediated by KC-MBON synaptic plasticity as well as some aspects of innate attraction mediated by other pathways.

## Discussion

In this study, we examined how PAM DANs, arguably the most well-studied “reward neurons” in the *Drosophila* brain, regulate feeding and other innate behaviors. Previous studies have addressed this question using various manipulations and behavioral assays and have obtained varying results.^10^^,^^13^^,^^19^^,^^20^^,^^21^^,^^22^^,^^23^^,^^24^^,^^25^^,^^26^^,^^39^^,^^40^ Our study builds on this previous work by 1) directly testing the innate valence of PAM neurons by measuring positional preference for optogenetic PAM activation, 2) comparing the acute effects of PAM activation in multiple behavioral assays, 3) testing the activation of the entire PAM population as well as broad or specific PAM subsets, and 4) testing the robustness of acute behavioral effects under different conditions (e.g., starvation or presence of odor).

We found that despite the ability of PAM activation to promote appetitive learning, activation of the PAM population evoked acute aversion in a variety of behavioral assays. PAM activation suppressed sugar feeding and proboscis extension, caused positional aversion, and drove aversive locomotor changes such as increased speed and turning. The aversive effects occurred even at low light intensities, suggesting that aversion is not due to excessive activation of the neurons. We were not able to identify subsets of PAM DANs that were sufficient to drive acute aversive responses, although most subsets were sufficient for appetitive learning, suggesting that aversion results from a subset that we have not identified or from the combined activation of multiple subsets.

These results demonstrate that the activation of the PAM population is perceived as an aversive stimulus despite conferring a rewarding value onto associated cues. Based on the known circuitry of the mushroom body, it is likely that these effects arise from distinct mechanisms: the direct activation of aversive MBONs causes acute aversion, whereas the depression of KC synapses onto the same aversive MBONs causes learned attraction to a paired odor. We found that PAM-mediated aversion occurred in either the presence or absence of odor-evoked activation of KC-MBON synapses, and PAM-mediated aversion could override learned attraction to an odor. Together, these findings provide new insight into the complex functions of PAM DANs and the opposing roles they play in different contexts.

### Relevance to natural behavior

A caveat of this study is its reliance on optogenetic activation. While neuronal activation is a well-established approach for determining whether specific neurons are sufficient to evoke a behavior,^41^ it is always possible that optogenetic activation does not recapitulate natural activity. This concern is particularly relevant when the results are counterintuitive, as in this study. Are there reasons to think that the aversive pathways engaged by optogenetic activation with *R58E02-Gal4* would not be engaged in response to natural stimuli that activate PAM DANs?

One possibility is that aversive behaviors result from activating PAM DANs at an abnormally high level. We do not believe this is the case because we consistently observed aversive responses across a wide range of light intensities, including very low intensities compared to those that cause phenotypes with other central brain neurons.^38^ Another possibility is that the use of continuous light to activate PAM DANs results in rapid compensatory changes in the circuit, but the fact that we observed the same behavioral effects with pulsed light (50 Hz) and that aversive behavioral changes occur within seconds argues against this idea. A third possibility is that natural stimuli do not normally activate the entire population of PAM DANs, implying that optogenetic stimulation causes an unnatural activity pattern. Because PAM responses to natural stimuli appear to be heterogeneous in their magnitude and time course,^22^^,^^29^^,^^42^ it is likely that optogenetic activation does not recapitulate the exact pattern of PAM activity. Moreover, different PAM subsets may drive different acute behavioral responses, as discussed below.

Finally, while it is possible that cell types other than PAM DANs may contribute to the phenotypes caused by activation with *R58E02-Gal4*, we consider it unlikely because this line is highly specific and well-validated. Immunostaining experiments from several labs,^8^^,^^9^ including images available in the Janelia FlyLight database,^43^ indicate that the only cells other than PAM neurons labeled in either the brain or ventral nerve cord are cells in the optic lobe that are reported to be glia,^8^ and thus are unlikely to contribute to the rapid behavioral effects we observed.

Neuronal silencing experiments represent a complementary approach to show that the natural activation of PAM DANs may promote aversive behaviors. This was the first approach that we took in our feeding experiments, and we found that optogenetic silencing of PAM DANs decreased sugar feeding. These results show that both increasing and decreasing PAM activity alter feeding behavior in the same direction, potentially representing an “inverted-U” model where activity within a certain range is optimal for the behavior and deviations in either direction cause behavioral dysregulation. Many behavioral effects of dopamine and other neuromodulators in mammals follow an inverted-U model.^44^^,^^45^^,^^46^ Moderate and high levels of neuromodulatory signaling can cause opposing effects on target neuron firing via the recruitment of inhibitory channels or circuits,^44^ or different levels of signaling can lead to different behavioral outcomes by altering the signal-to-noise ratio.^47^ Recordings of downstream neuronal activity would shed light on the mechanisms underlying the behavioral effects of PAM manipulations.

### Opposing functions of dopaminergic neurons in innate and learned behaviors

While most previous work on PAM DANs has focused on their role in appetitive learning, some studies have examined the role of these DANs in innate behaviors. Several experiments manipulating PAM DANs – either as a population or by targeting specific subsets – suggest a role in promoting innate behaviors related to reward seeking, such as food seeking,^19^^,^^20^ sugar feeding,^21^ water seeking,^10^ positional attraction,^39^ or tracking an appetitive odor,^22^ although other studies did not observe such effects.^13^^,^^40^ Based on these results, we expected that PAM activation would increase sugar feeding and were surprised to instead observe strong aversive effects on feeding and other behaviors.

During the course of our study, two new studies reported the effect of optogenetic PAM activation with *R58E02-Gal4* on immediate positional preference, similar to our experiments. The study by Rohrsen et al.^25^ observed positional aversion to red light in a T-maze assay, consistent with our results, although they did not observe the same effect when using yellow light (which is less effective at activating Chrimson) or in a single-fly Y-maze assay using closed-loop red light stimulation. In contrast, the study by Mohammad et al.^26^ reported that flies show positional preference for PAM activation using *R58E02-Gal4*. Although there are some technical differences between their study and ours, such as their use of linear rather than circular fly chambers, it is surprising that they observed consistent light preference across a range of intensities, whereas we observed consistent light aversion. While Mohammad et al. tested fed male flies rather than the food-deprived female flies that we used, we also observed aversion when testing male flies (Figure 2D) or fed flies (Figures S2H–S2J). We do not presently have an explanation for the difference between these results.

Aligned with our results, some previous work has hinted that PAM or protocerebral posterior lateral cluster 1 (PPL1) DANs, the counterparts of PAM DANs that mediate aversive learning,^1^^,^^7^ each have opposite roles in regulating innate versus learned behaviors. A study by Takemura et al. showed that optogenetic activation of a PPL1 subset elicited positional preference despite causing aversive olfactory learning.^24^ The same study found that reactivating subsets of PAM or PPL1 neurons after odor pairing, while flies are choosing between the CS+ and CS- odors, causes a decrease in the learned response,^24^ similar to our results – i.e., immediate DAN activation counteracts the effect of previously paired activation. This phenomenon has also been observed in larvae using broad PAM activation by *R58E02-Gal4* or activation of a single PAM neuron: pairing PAM activation with the odor causes future preference for the odor, but this preference is reduced if PAM DANs are reactivated during the test.^48^ The authors suggest that odor attraction represents a search for reward and PAM activation mimics the reward, thus causing the search to cease. However, this model would not explain our results showing that PAM activation alone elicits aversive responses.

Takemura et al. propose a related but broader model: the opposing relationship between the role of DANs in immediate behavior versus future learned behavior may reflect a need to compare current reward (encoded by DANs) and expected reward based on reward-associated cues that were previously learned (encoded by KC-MBON activation).^24^ This model implies that innate attraction to rewards must be mediated by circuits outside of the mushroom body, as the mushroom body circuits would tend to promote immediate aversion rather than attraction. This is known to be true in some cases; for example, sugar evokes locomotor suppression and positional preference through neurons located in the subesophageal zone rather than the mushroom body.^38^^,^^49^^,^^50^ One would expect the circuits for innate attraction to generally override PAM-mediated aversion, as sugar is innately attractive. We did not observe this result in our co-activation experiments, but this may be because genetic tools require us to activate sugar neurons and PAM DANs using the same light intensity, which may not reflect the natural balance of activity.

While multiple lines of evidence now suggest that at least some DANs have opposing roles in innate and learned behaviors, it is clear that there is heterogeneity in DAN function. Our study examined the role of three PAM subsets in feeding and five PAM subsets in learning, positional preference, and locomotion, finding that different subsets have different acute effects even though most of them cause appetitive learning. The fact that none of these subsets was sufficient to drive acute aversion suggests that aversion results from a subset that we did not test or from the combined activation of multiple subsets. In the latter case, aversion may arise from summing the effects across many DANs or from nonlinear interactions resulting from combined DAN activation. Recent studies show that different mushroom body compartments, containing specific DAN types and their target MBONs, do not simply function in parallel; these compartments may have excitatory or inhibitory interactions that could lead to emergent effects.^16^^,^^51^^,^^52^^,^^53^

The studies by Rohrsen et al.^25^ and Mohammad et al.^26^ tested a larger number of PAM subsets for positional preference and observed a range of behavioral responses, from attraction to aversion. It is not entirely clear how different PAM subsets could promote innate behaviors of opposing valence given that the behavioral response is thought to be determined by the balance between MBONs promoting attraction versus aversion, and PAM DANs target only the latter type.^7^ Perhaps different DANs have different effects on their MBON targets depending on the dopamine receptors expressed or other neurotransmitters that are co-released from the DANs.^54^ Mohammad et al. found that the acute effects of PAM activation depended on a combination of neurotransmitters released by DANs, including dopamine, glutamate, and octopamine.^26^ These authors also found that knocking down individual dopamine receptors (Dop1R1, Dop1R2, or Dop2R) in Kenyon cells or MBONs had only a mild to moderate impact on the acute effects of PAM activation, in contrast to the critical role of Dop1R1 in appetitive learning,^55^ suggesting that multiple receptors may contribute to immediate behavior.^26^ Given the strong acute aversion we observed with PAM activation, future studies could explore whether this aversion can be elicited by molecules that activate dopamine receptors or activate PAM targets through other mechanisms in *Drosophila* as well as other insect species, which could be exploited as a form of pest control.

The idea that mushroom body DANs in *Drosophila* have a dual role in modulating ongoing behavior and causing plasticity to modulate future behavior is strikingly similar to models of dopamine function in mammals.^2^^,^^3^ As in *Drosophila*, there is evidence that mammalian DANs are heterogeneous and co-release neurotransmitters in addition to dopamine,^3^ features that may contribute to the diverse functions of these neurons. Thus, the tools available in *Drosophila* to study the connectivity^56^^,^^57^ and function^41^ of individual neurons will facilitate a mechanistic understanding of the context-specific roles of DANs that will likely be relevant to mammalian systems.

### Limitations of the study

This study relies primarily on optogenetic activation to test the function of DANs, and the caveats associated with optogenetic activation are discussed above. Conflicting results between this study and another recent study^26^ have not yet been reconciled. Finally, while known circuitry and previous work on mushroom body function suggest that DANs promote acute aversion and appetitive learning through different actions on the same MBONs, this model remains to be confirmed.

## Resource availability

### Lead contact

Further information and requests for resources and reagents should be directed to and will be fulfilled by the lead contact, Anita Devineni (anita.devineni@emory.edu).

### Materials availability

This study did not generate new unique reagents.

### Data and code availability

• All data reported in this article will be shared upon request with the lead contact. Statistical results for all experiments can be found in Table S1.
• This article did not report new code. MATLAB code used for behavioral analyses will be shared upon request with the lead contact.
• Any additional information required to reanalyze the data reported in this article is available from the lead contact upon request.

## Acknowledgments

We thank members of the Devineni lab for input on the project, Yoshi Aso for help with behavioral setups, Adam Claridge-Chang for discussions about conflicting data, Jan Hawes for facilities assistance, and Chris Rodgers for comments on the paper. We thank Daisuke Hattori, the Janelia Research Campus, and the Bloomington Drosophila Stock Center (BDSC, supported by NIH P40OD018537) for providing fly stocks, and we acknowledge FlyBase for providing information about fly lines. This work was supported by the 10.13039/100001391Whitehall Foundation (grant 2022-08-017 to A.V.D.) and funding from the 10.13039/100006939Emory University SURE and LGS-SOAR programs.

## Author contributions

A.V.D. conceived and supervised the project, conducted proboscis extension assays, and assisted with some dissections and other experiments. F.V.L.-P., Y.C., R.V.J., J.Y., M.Y., and J.B. conducted all other experiments and analyzed the data. F.V.L.-P. generated initial drafts of the figures. A.V.D. generated the final figures and wrote the article with input from all authors. All co-authors approved the final version of the article.

## Declaration of interests

The authors declare no competing interests.

## STAR★Methods

### Key resources table

**Table:** 

REAGENT or RESOURCE	SOURCE	IDENTIFIER
**Antibodies**		

anti-DsRed (rabbit polyclonal)	Clontech	RRID: AB_10013483
anti-GFP (chicken polyclonal)	Aves Labs	RRID: AB_10000240
Alexa Fluor 568 (goat anti-rabbit polyclonal)	Life Technologies	RRID: AB_10563566
Alexa Fluor 488 (goat anti-chicken polyclonal)	Life Technologies	RRID: AB_2534096

**Chemicals, peptides, and recombinant proteins**		

all *trans*-retinal	Sigma-Aldrich	R2500

**Experimental models: Organisms/strains**		

*Drosophila, R58E02-Gal4*	Jenett et al. 2012^43^; Liu et al., 2012^8^	BDSC 41347
*Drosophila, R58E02-lexA*	Pfeiffer et al., 2010^58^	BDSC 52740
*Drosophila,* MB441B	Aso et al., 2014^37^	BDSC 68251
*Drosophila,* MB056B	Aso et al., 2014^37^	BDSC 68276
*Drosophila,* MB301B	Aso et al., 2014^37^	BDSC 68311
*Drosophila,* MB042B	Aso et al., 2014^37^	BDSC 68303
*Drosophila,* MB196B	Aso et al., 2014^37^	BDSC 68271
*Drosophila,* MB043C	Aso et al., 2014^37^	BDSC 68363
*Drosophila,* MB213B	Aso et al., 2014^37^	BDSC 68273
*Drosophila, Gr64f-Gal4*	Dahanukar et al., 2007^59^	BDSC 57669
*Drosophila, UAS-Chrimson-TdT*^*VK5*^	Duistermars et al., 2018^60^	N/A
*Drosophila, lexAop-CS-Chrimson*^*attp40*^	Axel lab	N/A

**Software and algorithms**		

MATLAB	Mathworks	www.mathworks.com
Fiji	Schindelin et al., 2012^61^	https://imagej.net/software/fiji/
GraphPad Prism, version 10	GraphPad Software	https://www.graphpad.com

### Experimental model and study participant details

#### Fly strains and maintenance

Flies were reared at 25°C on standard cornmeal/molasses food and maintained in constant darkness to avoid activation of optogenetic channels. Flies for behavioral experiments were collected within a few days of eclosion and transferred to food containing 1 mM all *trans*-retinal for 2–4 days prior to testing. Unless otherwise specified, behavioral experiments used flies that were food-deprived with water for one-day. Food deprivation is required in order to observe feeding in the optoPAD and is used in most studies of appetitive learning,^8^^,^^9^^,^^18^ and we wanted to keep conditions constant when testing the role of PAM DANs in different behavioral assays. During food deprivation, flies were housed in vials with 1 mM all *trans*-retinal solution on a piece of Kimwipe. All experiments used mated females except for the experiment shown in Figure 2D, which used males; similar effects were observed across sexes for this experiment. For other experiments, males were not tested so it is not possible to conclude whether there is an effect of sex.

Experiments using optogenetic activation or silencing compared experimental flies carrying both the Gal4 and UAS transgenes to *Gal4/+* and *UAS/+* flies carrying only one of the two transgenes. Genotypes used for each experiment are specified in the figures or legends, and detailed information about fly strains is provided in the key resources table. The PAM subsets labeled by each split-Gal4 line are based on annotations in the Janelia FlyLight Split-Gal4 database (https://splitgal4.janelia.org/) and in Aso et al.^37^

### Method details

#### optoPAD assay

##### Hardware and software

Assays using the optoPAD were conducted as previously described.^31^^,^^35^ The optoPAD was purchased from Pavel Itskov at Easy Behavior, who also provided code to run the assay using Bonsai and process the data using MATLAB. Detailed information regarding the design of the optoPAD and data processing methods can be found in Moreira et al.^31^ and Itskov et al.^32^ Briefly, each fly was placed in a 3 cm diameter circular arena made of transparent acrylic with two feeding sensors on the floor of the arena. Sucrose was mixed into 1% agarose and pipetted into the center of each feeding sensor; the sucrose concentration for each experiment is specified in the figures and text. Each sensor contains two electrodes, an inner “food” electrode and an outer “fly” electrode. Feeding events are detected as capacitance changes when the fly touches both electrodes. Optogenetic stimulation is delivered using LEDs positioned above each arena (625 nm for red and 523 nm for green). LED intensity can be varied by controlling the input voltage. We measured LED power output using a power meter (Thorlabs, PM121D); intensities for each experiment are specified in the figures and text. Real-time detection of feeding events and closed-loop LED stimulation are controlled by Bonsai. Real-time detection of feeding events relies on quantifying a running difference of consecutive capacitance values and applying a finite impulse response filter that is then thresholded.^31^ The onset of a continuous light pulse was triggered immediately upon detection of an interaction with the specified food source, and the light remained on for 1.5 s. Because feeding initiation triggers light onset, this assay may elicit operant conditioning even though this was not the primary goal. Following each experiment, raw capacitance data were processed using the MATLAB code provided by Easy Behavior, described in Itskov et al.,^32^ which filters the capacitance signal to remove slow fluctuations in the signal, then takes the root-mean-square in consecutive windows, then thresholds this signal to extract feeding activity bouts. The output of this code generates files containing the number and duration of feeding activity bouts during the assay as well as the duration of feeding bout activity over time in 10 s bins.

##### Assay conditions

Individual flies were loaded into each optoPAD chamber using aspiration. 24 flies were tested in parallel, and data recording was started as soon as all flies were loaded (typically ∼5 min). Assays were conducted for 1 h in the dark at room temperature (∼23°C) in a dedicated behavior room.

#### Associative learning, positional preference, and locomotor assays

##### Behavioral arena

Assays for associative learning, positional preference, and locomotion were conducted using previously described methods.^35^^,^^38^ The behavioral arena was originally built based on designs from the Janelia Research Campus and is described in Aso et al.^34^ and Aso & Rubin.^18^ Detailed documentation regarding the hardware specifications and design files is available from Janelia (arena: https://www.janelia.org/open-science/combined-infrared-and-optogenetic-led-panel; olfactory delivery: https://www.janelia.org/open-science/four-way-olfactometer-arena-fruit-flies). The arena is a 10 cm diameter circular chamber with a glass cover, and flies are filmed from above using a USB camera (Point Gray, #FL3-U3-13Y3M-C) with a 25 mm focal length lens (Edmund Optics, #63–246). Flies were filmed at 30 frames/s. The infrared (850 nm) light for illumination and the red (627 nm) LED array for optogenetic stimulation are located just beneath the chamber. Each quadrant of the arena has an inlet for airflow, allowing for odor delivery, and air is removed through a vacuum port at the center of the arena. Airflow was used for learning assays and other experiments using odors, but was not turned on for locomotion or positional preference assays in the absence of odor. Video acquisition, optogenetic stimulation, and odor delivery were synchronized using MATLAB code provided by Janelia.

##### Assay conditions

20–25 flies were tested per trial. Flies were loaded into the chamber using aspiration and were given ∼3 min to habituate before the experiment started, followed by a 60 s recording of baseline behavior in the absence of any stimuli. Assays were performed at room temperature (∼23°C) in a dedicated behavior room.

##### Odor delivery

400 mL/min air was split into two streams that each flowed into one pair of opposing quadrants. Each stream was independently odorized by flowing air through bottles containing odor dilutions, then was split again to provide input to the two opposing quadrants, representing a flow rate of 100 mL/min air into each quadrant. A vacuum line removed air from the center of the arena at a flow rate of 400 mL/min. The odors used were 3-octanol (OCT, 1:1000) and 4-methylcyclohexanol (MCH, 1:750). Odorants were diluted into mineral oil and 2 mL of each odorant solution (or mineral oil as a control) was pipetted onto a piece of thick filter paper (Thermo Fisher Scientific, #88615) placed inside of a small glass bottle (Cole-Parmer, #EW-99535-16). The flow of air into the control or odorant bottles was regulated using solenoid valves (Lee Co #LFAA0503110H).

##### Associative learning protocols

First, the CS+ odor was presented in all quadrants for 1 min along with light stimulation (30 × 1 s pulses with a 1 s interpulse interval) that started 5 s after odor valve opening, following the protocol used in previous learning studies.^18^ After a 1 min rest, the CS- odor was presented alone for 1 min. Following another 1 min rest, the CS+ and CS- odors were simultaneously delivered to different sets of opposing quadrants for 1 min, allowing the flies to choose between the odors. After another 1 min rest, the two odors were presented again for 1 min but the odor quadrants were switched to control for any spatial bias. Which odor was used as the CS+ or CS- was counterbalanced across trials. 60% light intensity (30 μW/mm^2^) was used for learning experiments. Learning experiments with light stimulation during the odor test (Figure 6E) used 50 Hz pulsed light (10 ms pulse width) at 40% intensity presented simultaneously with the odors. Pulsed light was used for this experiment because the light had to remain on for 60 s (the duration of the odor test) rather than 30 s (the usual duration of preference experiments) and we wanted to avoid potential issues with prolonged continuous light stimulation.

##### Locomotion and positional preference assays

For most experiments, locomotion and positional preference were assayed sequentially in the same flies. To quantify locomotor effects, light stimulation was presented for 5 s. In general, each of the three light intensities (10%, 40%, and 70%, corresponding to 4, 20, and 35 μW/mm^2^, respectively and measured in a previous study^35^) was delivered to the same flies in increasing order of intensity, with at least 2 min between stimulations to ensure that the behavior had recovered. After an additional rest period, the same flies were then tested for positional preference by delivering 10% light stimulation to two opposing quadrants for 30 s. The flies then had a 30 s rest period without light, followed by 10% light stimulation in the other two quadrants for 30 s. Switching the light quadrants ensured that we could assess light preference independently of any spatial bias. This protocol was then repeated sequentially with 40% and 70% light. A lower intensity (6%, close to the threshold of the lowest intensity stimulation our setup is capable of delivering) was separately tested for *R58E02-Gal4* activation. Experiments testing locomotion and positional preference in the presence of a background odor (Figures 6B–6D) used OCT or MCH at the same concentrations and flow rates described for learning, and these experiments included only two sequential tests using 40% light intensity (for locomotor assays, the same flies were tested sequentially with each of the two odors; for choice assays, the same flies were tested sequentially with the odor presented in different quadrants, and only one odor was tested for each set of flies). Continuous light stimulation was used for all locomotor and preference experiments other than the pulsed light experiments using 50 Hz stimulation with a 10 ms pulse width.

##### Video analysis

Fly videos were analyzed using FlyTracker,^62^ which quantified the position, forward velocity, and angular velocity of each fly at each time point. FlyTracker output was further analyzed in MATLAB to quantify light-induced locomotor changes or preference for the light or odor quadrants. Preference index (PI) values were quantified in 1 s bins. For positional preference, PI was calculated as (# flies in light quadrants – # flies in non-light quadrants)/total # flies. For learning, PI for the CS+ was calculated as (# flies in CS+ quadrants – # flies in CS- quadrants)/total # flies. Forward and angular velocities were averaged over 10-frame bins (0.33 s) except for the zoomed-in plots shown in Figures 6F and S5D. To quantify locomotor changes evoked by light stimulation, we averaged forward or angular velocity across all flies during the 5 s light presentation and subtracted these values from the baseline values averaged over the 4 s preceding light onset. Quantifying the light-evoked change in locomotion was more meaningful than quantifying absolute velocity during light because different groups may show different baseline velocities. For statistical analyses of locomotion, preference, and learning data, each trial was considered to be a single data point (“n”).

#### Proboscis extension assays

##### Assay description and fly preparation

Proboscis extension assays were conducted as previously described.^63^^,^^64^ Assays were performed at room temperature (∼22°C). Flies were anesthetized on ice and immobilized on their backs using myristic acid wax. The two anterior pairs of legs were glued down so that the proboscis was accessible for stimulation. Flies recovered from gluing for 30–60 min in a humidified chamber and were water-satiated before testing.

##### Taste stimulation

100 mM sucrose was used as the sugar stimulus and was delivered using a Kimwipe wick.^65^ The stimulus was manually delivered to the fly’s labellum for less than 1 s and was retracted before the fly could consume it. First, each fly was given 5 consecutive trials, separated by a few seconds, without light stimulation. Flies were then allowed to rest for at least 5 min before being water-satiated again and then being tested with 5 consecutive trials with simultaneous light stimulation, using a red LED (617 nm, Luxeon) with a 29.8° frosted lens placed 6.5 cm from the fly. The power output at the fly was measured to be 42 μW/mm^2^. The LED was manually turned on (using a pushbutton controlled by an Arduino) just before the sugar stimulation trials and was turned off immediately after sugar stimulation.

##### Data recording and analysis

We recorded the presence or absence of proboscis extension on each trial, and only full extensions were counted. For each fly, we quantified the percentage of trials evoking extension for each condition, and we then calculated the difference in this percentage for light versus non-light conditions. Flies were also tested again without light stimulation at least 5 min after the light stimulation trials and showed similar response levels as in the first set of non-light trials, but these data were not included in the paper because they could be confounded by learning effects resulting from pairing PAM activation with sugar.

#### Immunostaining

Immunostaining experiments were performed as previously described.^28^^,^^35^ Briefly, brains were dissected in phosphate buffered saline (PBS), fixed for 15–20 min in 4% paraformaldehyde, washed multiple times with PBS containing 0.3% Triton X-100 (PBST), blocked with 5% normal goat serum for 1 h, incubated with primary antibody at 4°C for 2–4 days, washed in PBST, incubated with secondary antibody at 4°C for 1–2 days, washed in PBST and PBS, and mounted in Vectashield. For staining of Gal4 or split-Gal4 lines with *UAS-Chrimson*, we used rabbit anti-DsRed (1:500) as the primary antibody and Alexa Fluor goat anti-rabbit 568 (1:500) as the secondary antibody. For *R58E02-lexA* staining with *lexAop-Chrimson*, we used chicken anti-GFP (1:1000) as the primary antibody and Alexa Fluor goat anti-chicken 488 (1:500) as the secondary antibody. Images were acquired on an Olympus IX81 spinning-disk confocal microscope and were processed using Fiji software.

### Quantification and statistical analysis

Statistical analyses were performed using GraphPad Prism, Version 10. Statistical tests and results are reported in the figures and figure legends, and detailed reporting of all statistical results can be found in Table S1. We used one-way ANOVA followed by Dunnett’s test when comparing behavioral metrics for experimental flies to *Gal4/+* and *UAS/+* controls, as Dunnett’s test is a commonly used method for comparing a single group to all other groups without needing to perform all possible pairwise comparisons, whereas we used Bonferroni’s post-test to perform all pairwise comparisons (Figures S5A–S5C) or to compare selected groups when there was more than one experimental group present (Figure 6F). We used two-way ANOVA followed by Bonferroni’s post-tests when comparing metrics for control and optogenetic conditions for each genotype, as this post-test compares metrics within each group. When comparing genotypes, positive effects are only reported if experimental flies show a significant difference from both controls, with a significance threshold of *p* < 0.05. All graphs represent mean ± standard error of the mean (SEM). Sample sizes are listed in the figure legends. For all graphs, ∗∗∗∗*p* < 0.0001, ∗∗∗*p* < 0.001, ∗∗*p* < 0.01, ∗*p* < 0.05, ns = not significant (*p* > 0.05).

## Supplemental information

Document S1. Figures S1–S6

Table S1. Summary of all statistical results in this study
