# Supplementary material for: Dual roles of Drosophila reward-encoding dopamine neurons in regulating innate and learned behaviors
Source: iScience. 2025 Oct 23;28(11):113817. doi: 10.1016/j.isci.2025.113817 (PMC12661156; doi:10.1016/j.isci.2025.113817)
Supplement: Document S1. Figures S1–S6 [file mmc1.pdf]

iScience, Volume *28*

Supplemental information

Dual roles of *Drosophila* reward-encoding dopamine neurons in regulating innate and learned behaviors

Fiorella V. Lozada-Perdomo, Yuzhen Chen, Ruby V. Jacobs, Joyce Yeo, Meifeng (Maia) Yang, Janhavi Bhalerao, and Anita V. Devineni

Feeding bouts over time: representative examples

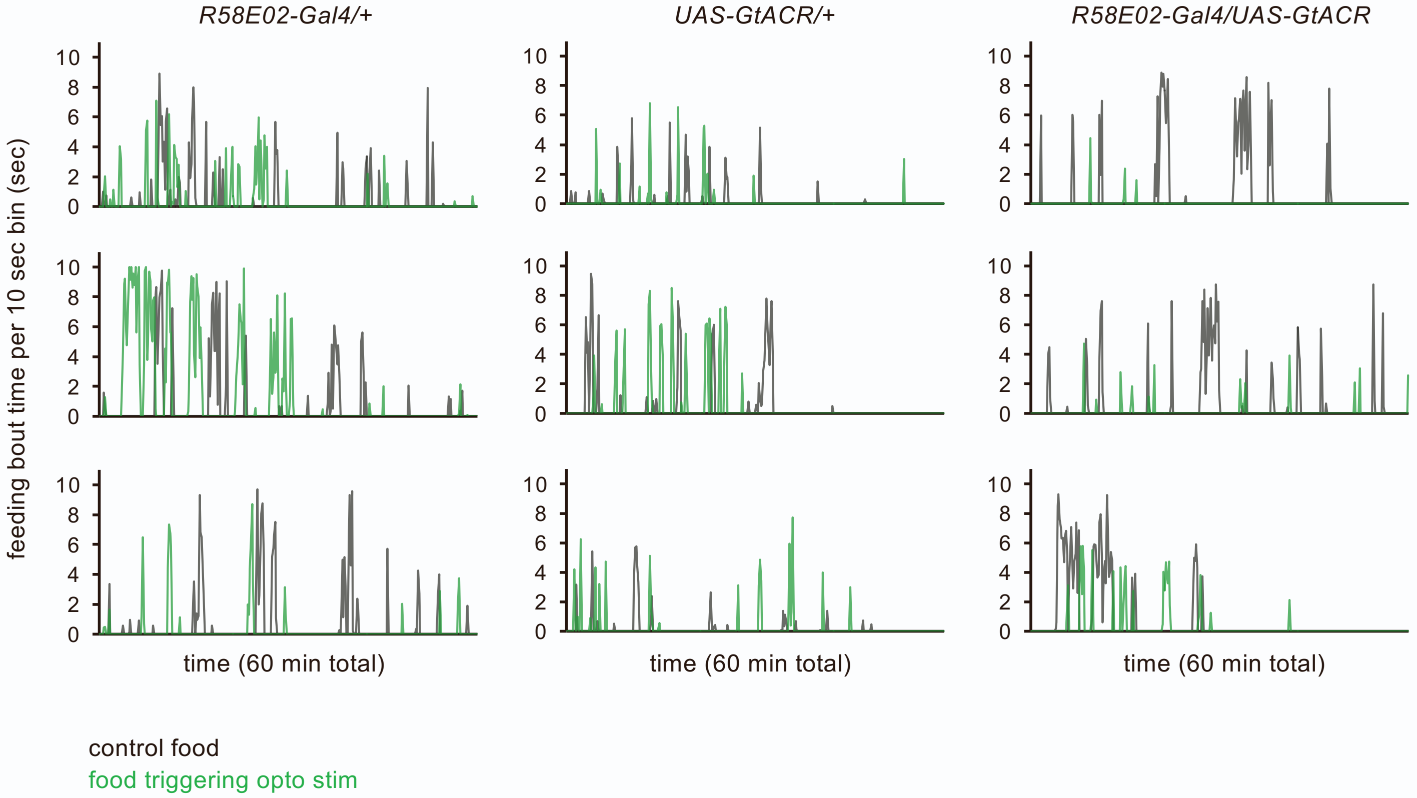

Figure S1, related to Figure 1. Representative examples showing timecourse of feeding activity in the optoPAD

Representative examples of feeding activity over time for flies with PAM silencing (*R58E02-Gal4/UAS-GtACR*) and control flies (*R58E02-Gal4/+* and *UAS-GtACR/+*). Data are from the same experiment shown in Figure 1E-F. For each genotype, graphs show feeding bouts over time for 3 individual flies (rows). The graphs show the duration of feeding bout activity in 10 sec bins over the 60 min assay, plotted separately for the control and optogenetic-linked food sources. For most flies, feeding preferentially occurs in the first half of the assay (because flies become satiated over time), and bouts of feeding on different food sources can be either intermingled or segregated in time. Experimental flies tend to show less feeding on the food triggering optogenetic stimulation, although this is not apparent in every fly.

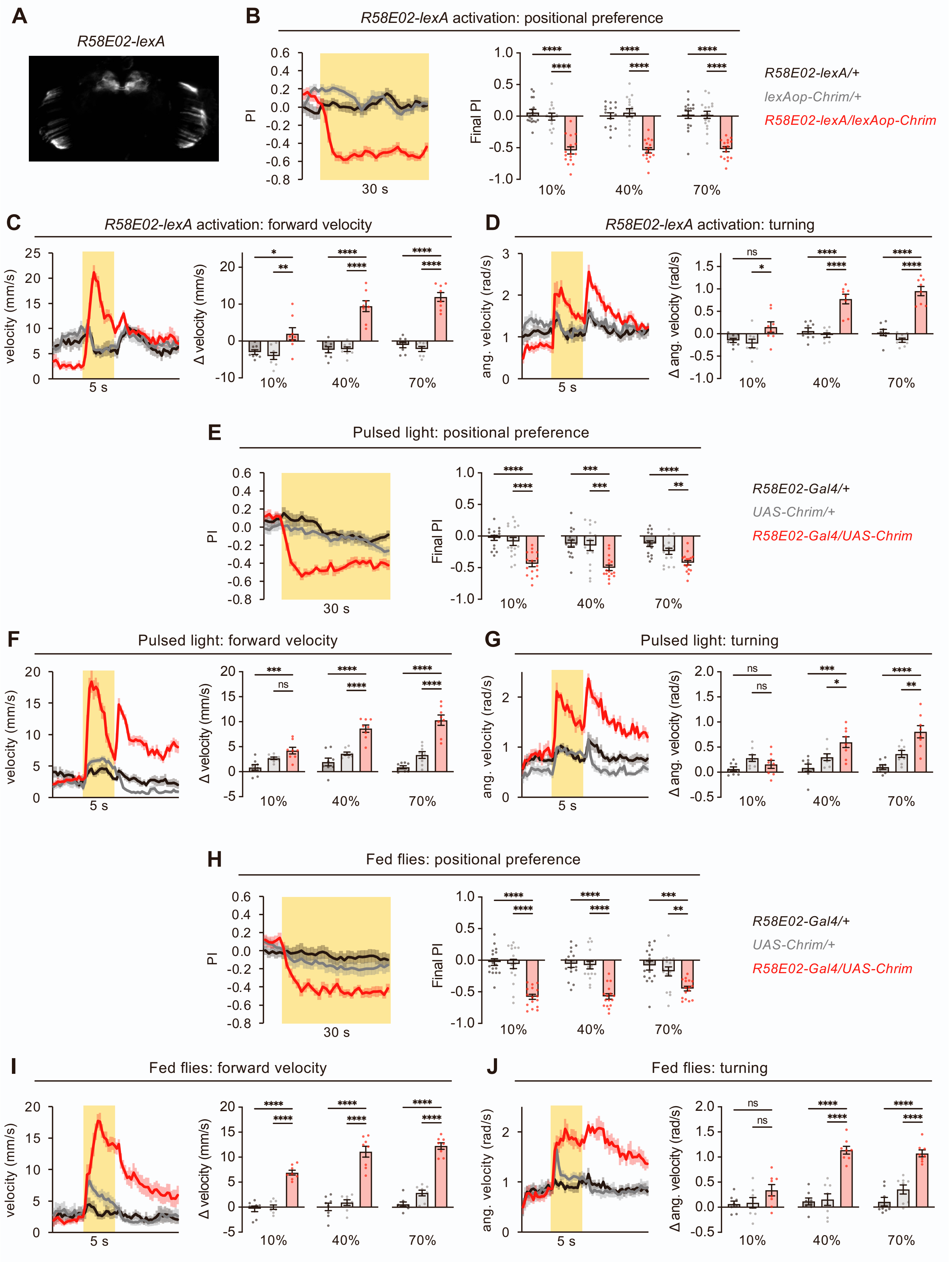

A
R58E02-lexA
B
R58E02-lexA activation: positional preference
PI
30 s
Final PI
10%
40%
70%
R58E02-lexA/+
lexAop-Chrim/+
R58E02-lexA/lexAop-Chrim
C
R58E02-lexA activation: forward velocity
velocity (mm/s)
5 s
Δ velocity (mm/s)
D
R58E02-lexA activation: turning
ang. velocity (rad/s)
Δ ang. velocity (rad/s)
E
Pulsed light: positional preference
R58E02-Gal4/+
UAS-Chrim/+
R58E02-Gal4/UAS-Chrim
F
Pulsed light: forward velocity
G
Pulsed light: turning
H
Fed flies: positional preference
I
Fed flies: forward velocity
J
Fed flies: turning

Figure S2, related to Figure 3. Additional experiments showing that PAM activation causes acute aversion

(A) Expression pattern of *R58E02-lexA* driving *lexAop-Chrimson*.

(B-D) PAM activation using *R58E02-lexA* driving *lexAop-Chrimson* caused positional aversion (B) and an increase in forward velocity (C) and turning (D), consistent with results using *R58E02-Gal4* (Figure 3D-I).

(E-G) PAM activation using *R58E02-Gal4* driving *UAS-Chrimson* with 50 Hz pulsed light activation caused positional aversion (E) and an increase in forward velocity (F) and turning (G), consistent with results using continuous light (Figure 3D-I).

(H-J) PAM activation using *R58E02-Gal4* driving *UAS-Chrimson* in fed flies caused positional aversion (H) and an increase in forward velocity (I) and turning (J), consistent with results using flies starved for one day (Figure 3D-I).

In all panels: Line graphs show behavior over time at 70% intensity (n = 16 trials, 8 sets of flies for preference; n = 8 sets of flies for locomotion). Bar graphs show final PI over the last 5 sec (B, E, H) or change in forward or angular velocity (C-D, F-G, I-J) during the light period compared to the pre-light baseline. Genotypes were compared using one-way ANOVA followed by Dunnett's test. ****$p<0.0001$, ***$p<0.001$, **$p<0.01$, *$p<0.05$, ns = not significant ($p>0.05$).

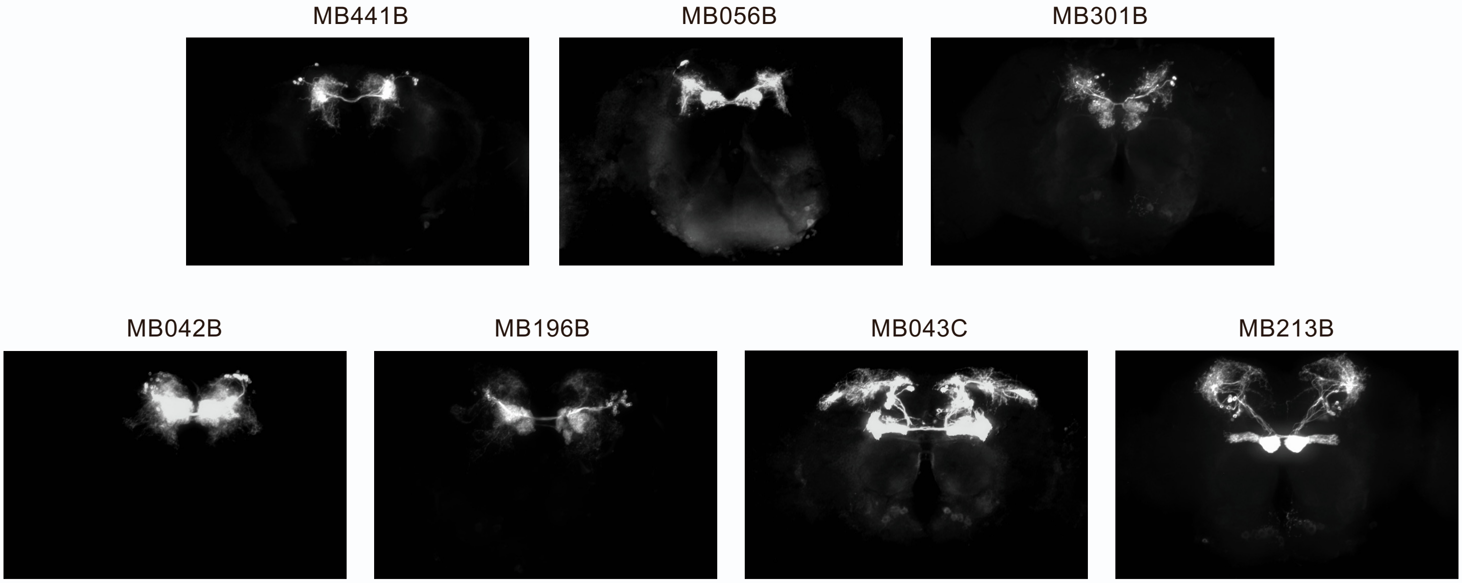

Figure S3, related to Figures 4 and 5. Expression patterns of split-Gal4 lines labeling PAM subsets

Expression patterns of split-Gal4 lines driving expression of *UAS-Chrimson*.

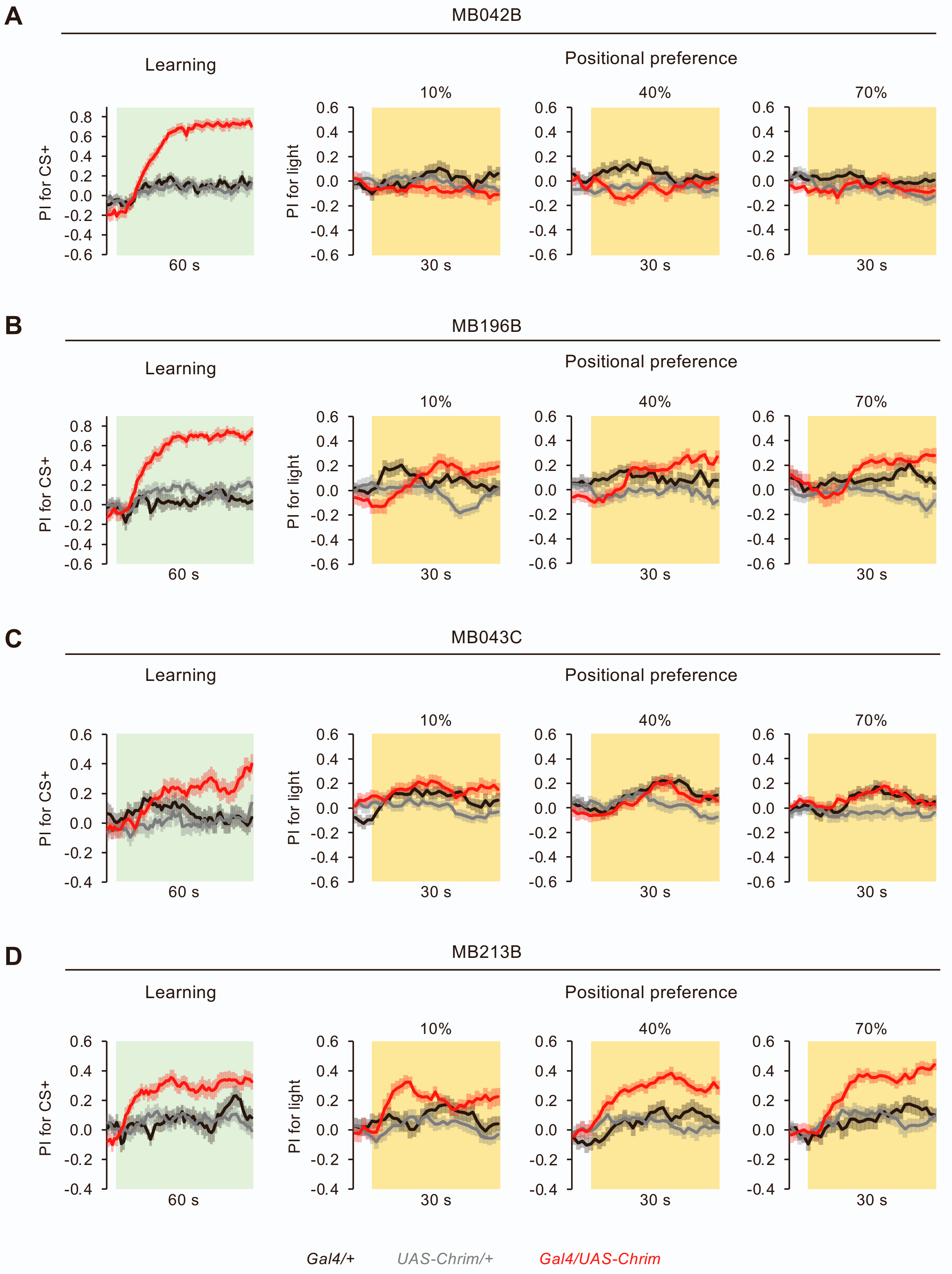

Figure S4, related to Figure 5. Preference values over time for activation of PAM subsets

Graphs showing behavior over time for the experiments shown in Figure 5. Left graphs show PI for the CS+ during learning assays. Right graphs show PI for the light quadrants during positional preference assays. Sample sizes are 14-24 trials (7-12 sets of flies) for learning and 22-24 trials (11-12 sets of flies) for positional preference.

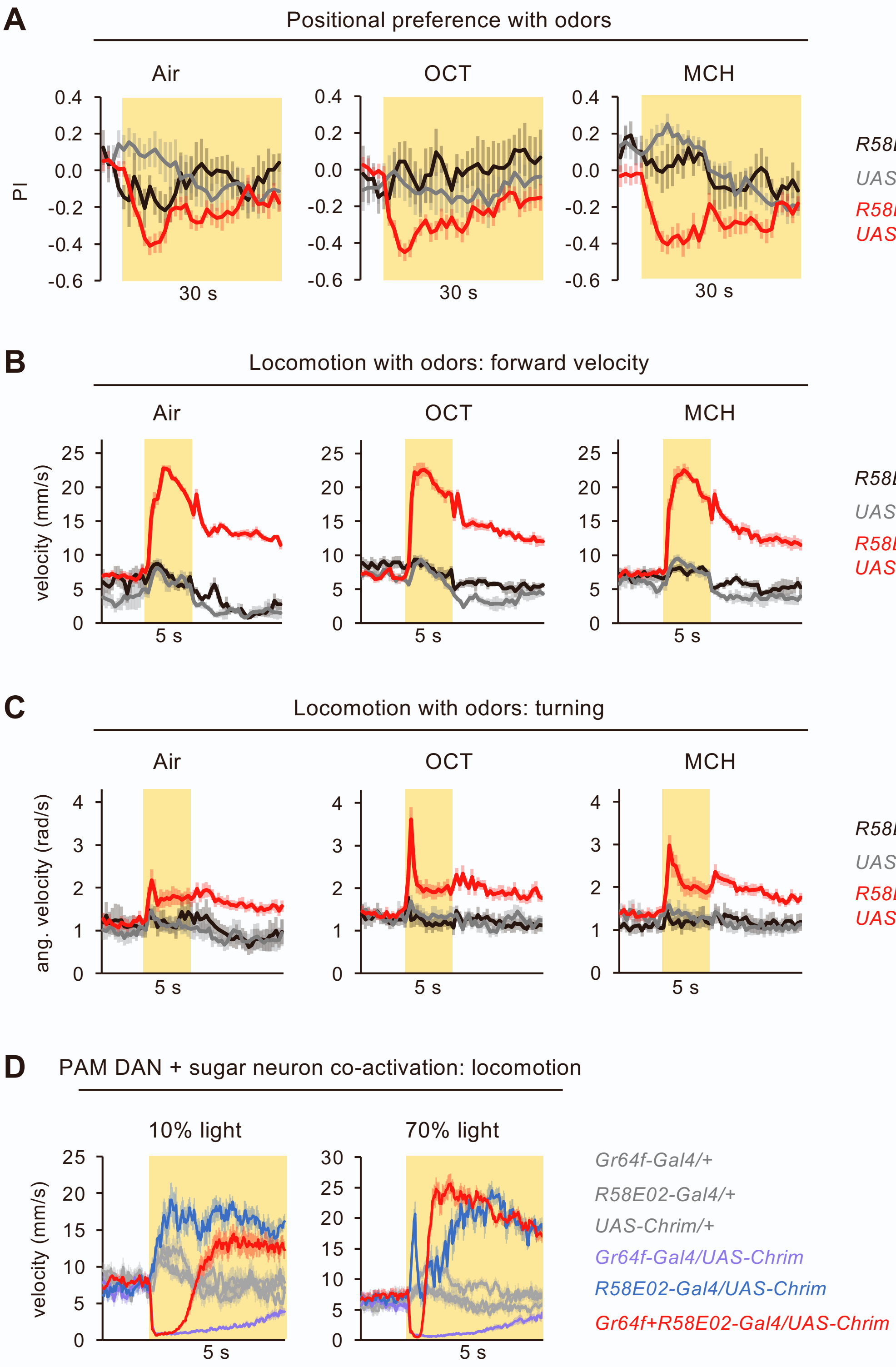

Figure S5, related to Figure 6. Additional data for PAM activation with odors or sugar neuron co-activation

(A-C) Results from PAM activation using *R58E02-Gal4* in the presence of odors (OCT or MCH) or airflow only. These are the same experiments presented in Figure 6B-6D but with data for each odor shown separately. 40% light was used. (A) n = 6-10 trials, 3-5 sets of flies for control genotypes and n = 36 trials, 18 sets of flies for the experimental genotype. (B-C) n = 4-6 sets of flies for control genotypes and n = 8-10 sets of flies for experimental genotypes. In panels A-C, the experimental genotype did not show a significant difference in PI or light-evoked change in velocity across the air, OCT, and MCH conditions (one-way ANOVA followed by Bonferroni's post-tests).

(D) Effects of co-activating PAM neurons (*R58E02-Gal4*) and sugar-sensing neurons (*Gr64f-Gal4*) on locomotion at 10% and 70% intensity (n = 7-13 sets of flies). Velocity graphs are zoomed in and not smoothed, as in Figure 6F, in order to show the transient stopping of flies with co-activation.
